# How does artificial intelligence improve ophthalmology education outcomes?—The mediating role of learning motivation and self-efficacy

**DOI:** 10.3389/fpsyg.2026.1759662

**Published:** 2026-04-02

**Authors:** Xia Wang, Siyan Jin, Ping Hu, Hai Jiang

**Affiliations:** Ophthalmologic Center of the Second Hospital, Jilin University, Changchun, China

**Keywords:** artificial intelligence, artificial intelligence literacy, learning motivation, ophthalmology student, self-efficacy

## Abstract

**Background:**

Artificial intelligence (AI) is increasingly being applied in medical education, yet evidence on how it translates into achievement it is most effective remains limited in ophthalmology.

**Methods:**

This cross-sectional study examined undergraduate ophthalmology students at a university in Jilin Province, China. A total of 416 students were randomly selected at two time points—November 2024 and March 2025—corresponding to the autumn 2024 and spring 2025 course cohorts, respectively. Survey measures captured AI usage, learning motivation, self-efficacy, final examination scores, and AI literacy. A moderated parallel mediation model was tested using structural equation modeling.

**Results:**

AI usage was positively associated with academic performance (*β* = 0.247, *p* < 0.001). Indirect effects via learning motivation and self-efficacy were significant, with standardized effects of 0.081 and 0.079, with confidence intervals of 0.059 to 0.103 and 0.017–0.141, respectively. AI literacy strengthened the path from AI usage to learning motivation, interaction (*β* = 0.183, *p* < 0.001).

**Conclusion:**

AI usage is associated with higher ophthalmology academic performance partly through increased learning motivation and self-efficacy, and AI literacy strengthened the AI usage in the learning motivation path. Given the cross-sectional design, longitudinal validation is warranted.

## 1 Introduction

The integration of artificial intelligence (AI) into higher education is revolutionizing pedagogical approaches, offering unprecedented opportunities for personalized learning and instructional support (Zawacki-Richter et al., 2019). In the demanding field of medical education, particularly in ophthalmology, which requires the synthesis of complex theoretical knowledge and precise practical skills, AI-powered tools such as virtual patient simulators, adaptive learning platforms, and image recognition systems hold significant promise for enhancing learning outcomes (Oshika, 2025; Wang et al., 2025; Muntean et al., 2023). However, while development in AI grows, a critical question remains: what are the precise psychological mechanisms through which AI utilization is associated with improved academic achievement?

Existing research has primarily focused on the direct correlation between the use of technologies such as AI and academic performance, often overlooking the underlying motivational processes (Chang et al., 2022; Feigerlova et al., 2025; Hayat et al., 2020). According to Social Cognitive Theory, learning motivation, which drives the direction and intensity of goal-oriented behavior, and self-efficacy, which refers to an individual’s belief in their capability to organize and execute courses of action to attain goals, are two pivotal personal factors (Locke, 1987). Preliminary studies suggest that AI can provide timely feedback and scaffolded learning, potentially boosting students’ confidence and engagement (Keller, 2016). Large structural equation models show that motivation and self-efficacy influence academic performance both directly and indirectly through greater learning engagement (Wu et al., 2020). These studies position motivation and self-efficacy as proximal drivers of academic success in medical training, implying that AI may raise achievement partly by strengthening motivation and self-efficacy rather than by simply transmitting declarative knowledge (Bai and Wang, 2025). Yet, the interplay between AI usage, these psychological constructs, and concrete academic outcomes in a specific domain like ophthalmology is not well understood. This mediating pathway has rarely been tested with objective academic outcomes in a concrete ophthalmology course.

However, students vary in AI literacy, defined as the set of competencies that allows a learner to understand how AI works, critically evaluate its output, communicate and collaborate with AI tools, and apply them responsibly in authentic tasks. AI literacy therefore, reflects not only technical skill but also confidence in managing AI feedback (Si, 2025; Long and Magerko, 2020).

Therefore, the present study addresses this gap by examining whether AI usage during an undergraduate ophthalmology module is associated with end-of-term examination performance, whether learning motivation and self-efficacy operate as parallel mediators in this relationship, and whether AI literacy moderates these effects. This investigation will delve into how AI tools are associated with to educational success, offering valuable insights for educators and instructional designers aiming to leverage AI not just as an information tool but as a catalyst for fostering the psychological drivers of academic excellence.

## 2 Hypothesis development

### 2.1 The impact of artificial intelligence on ophthalmology education outcomes

The application of artificial intelligence technology has significantly enriched the resource supply for ophthalmology education, enhancing the visualization and interactivity of teaching content. Traditional ophthalmology instruction often relies on instructor narration and static images. In contrast, AI-driven intelligent image recognition and case analysis systems can present multidimensional pathological images in real time, simulate disease progression, and algorithmically annotate typical pathological features. This enables students to grasp complex pathophysiological mechanisms within a dynamic, visual environment (Kang et al., 2024). For instance, AI-assisted retinal image recognition systems help students rapidly understand the imaging manifestations of common diseases like macular degeneration and diabetic retinopathy, significantly improving the accuracy of their clinical judgments (Wang et al., 2025). This pedagogical shift transforms the learning process from passive reception to active exploration, providing students with a more inspiring learning experience.

Secondly, artificial intelligence promotes adaptive optimization of student learning behaviors in ophthalmology education by establishing personalized learning pathways and real-time feedback mechanisms. Students receive immediate AI feedback during tasks such as retinal image recognition, case analysis, and diagnostic reasoning, enabling them to continuously refine their cognitive structures through error correction. Furthermore, the traceable learning data provided by AI systems allows instructors to analyze teaching weaknesses, thereby achieving scientific and precise teaching strategies (Mastour et al., 2025).

Furthermore, AI-driven virtual simulation technology and generative modeling further expand the practical dimension of ophthalmology teaching (Heinke et al., 2024). Through the combination of virtual reality, augmented reality, and AI simulation systems, students are able to complete ophthalmic operation training and diagnostic decision-making exercises in virtual operating theatres or virtual clinic environments, thus obtaining high-intensity clinical skills training under low-risk conditions (Fazlollahi et al., 2022).

Taken together, prior research suggests that AI can scaffold learning in vision science and clinical ophthalmology, improve procedural skill acquisition, and deliver individualized academic support. Thus, in the present study, we expect a direct, positive association between AI usage and objective achievement in ophthalmology.

*H1*. Students’ level of AI usage during the undergraduate ophthalmology module is positively associated with their final exam scores.

### 2.2 The mediating role of learning motivation and self-efficacy

Learning motivation and academic self-efficacy are well-established psychological resources that influence the intensity with which students engage with challenging material, their persistence, and their ability to self-regulate effectively. Studies in health professions education show that higher motivation predicts greater learning engagement and better academic outcomes among medical trainees (Wu et al., 2020). There is growing empirical support that AI-based instructional supports can strengthen exactly these proximal drivers. Xu et al. (2025) examined over one thousand university students and found that AI-driven personalized feedback increased goal clarity, boosted self-efficacy, and heightened learning engagement; in turn, these motivational gains predicted better goal attainment and academic outcomes. In other words, AI systems that deliver timely, individualized, actionable feedback do more than transmit information. They also make learners feel more competent and more invested, which are classic antecedents of persistence and achievement in demanding content areas. Experimental evidence from surgical simulation further supports this pathway. Medical students trained with an AI tutor not only outperformed peers taught by human experts but reported affective and cognitive states (for example, confidence and manageable cognitive load) comparable to expert-led instruction, suggesting that AI feedback can sustain motivation and perceived capability even in high-stakes technical tasks (Fazlollahi et al., 2022).

Both constructs, therefore, provide plausible proximal drivers linking instructional environments to final performance. On this basis, we argue that the effect of AI usage on final achievement will be indirect, operating through higher learning motivation and stronger self-efficacy.

*H2a*. Learning motivation mediates the relationship between AI usage and final exam score in ophthalmology.

*H2b*. Academic self-efficacy mediates the relationship between AI usage and final exam score in ophthalmology.

### 2.3 The moderating role of Al literacy

On this basis, this study further introduces artificial intelligence literacy as a moderating variable. AI literacy refers to an individual’s comprehensive ability in understanding, applying and evaluating AI systems (Chiu et al., 2024). Students possessing higher levels of AI literacy tend to better comprehend the functions and principles of AI technology in teaching, thereby demonstrating greater initiative and confidence in their learning process (Long and Magerko, 2020). In medical teaching, students with higher AI literacy are able to make fuller use of the diagnostic assistance, image analysis and personalized feedback provided by AI platforms, which is more likely to stimulate their interest and confidence in learning; on the contrary, those with lower AI literacy may have lower motivation and self-efficacy due to a lack of understanding of the technology or barriers to its use (Laupichler et al., 2024). Therefore, this study hypothesizes that AI literacy has a significant moderating effect between AI usage and the mediating variables, i.e., the higher the level of AI literacy, the stronger the facilitating effect of AI usage on learning motivation and self-efficacy.

*H3a*: Artificial intelligence literacy moderates the relationship between AI usage and learning motivation, i.e., when the level of AI literacy is high, the positive effect of AI usage on learning motivation is stronger.

*H3b*: Artificial Intelligence Literacy moderates the relationship between Artificial Intelligence usage and self-efficacy, i.e., when the level of Artificial Intelligence Literacy is high, the positive effect of Artificial Intelligence use on self-efficacy is stronger.

**Figure 1:**
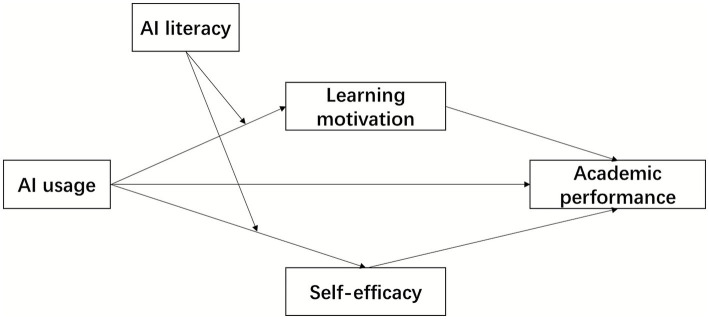
Hypothetical model of the dual mediating effect.

The model diagram is shown in Figure 1.

## 3 Materials and methods

### 3.1 Study population and procedure

The study followed an observational cross-sectional design. We randomly selected 416 undergraduate students from a university in Jilin province, China, in November 2024 and March 2025 to participate in an ophthalmology teaching course in the autumn semester and spring semester of 2024 and 2025, respectively. Ethical approval was waived by the Ethics Committee of Jilin University. All participants provided written informed consent.

Before official grade release, students were informed of the purpose of the study and were asked to provide written informed consent. Participation was voluntary and had no impact on course standing, assessment, or evaluation by faculty. Students who consented completed a structured questionnaire that assessed four domains: (1) AI usage; (2) Learning motivation; (3) Self-efficacy, (4) AI literacy. All questionnaire items were administered in the classroom setting using a secure online survey link or paper form. Finally, 408 valid questionnaires were recovered. To enable linkage with objective performance while preserving confidentiality, each participant generated a unique study code that could be matched by an independent teaching administrator to the final examination score. Inclusion criteria were as follows: (1) they were officially enrolled in the required undergraduate ophthalmology module during the target semester. (2) They provided written informed consent to participate in the study. Exclusion criteria included (1) they were visiting, exchange, or short-term elective students who attended selected ophthalmology sessions but were not assessed through the standard final examination of the module. (2) They were repeating the module due to prior course failure. (3) They were absent from the final examination or received an incomplete or deferred grade.

For clarity, in this study, “AI usage” specifically referred to students’ engagement with (a) generative AI chatbots for study support (e.g., ChatGPT), (b) AI-enabled virtual ophthalmic simulation modules for skills rehearsal (e.g., slit-lamp/fundoscopy or basic surgical scenarios), (c) adaptive learning/recommendation features embedded in the learning management platform (e.g., algorithm-driven quiz selection and personalized feedback), and (d) AI-assisted image interpretation tools used in teaching (e.g., automated retinal image analysis).

### 3.2 Measurement

#### 3.2.1 AI usage (AU)

Four questions were used to assess students’ use of AI on a 5-point Likert scale, where 1 means “strongly disagree” and 5 means “strongly agree” (Medcof, 1996). The questions were as follows: (1) I spend more and more time interacting with AI during my coursework. (2) I use AI to seek answers to questions I have during my coursework. (3) I use AI to ask for advice on course tasks. (4) I am becoming more and more interested in AI tools.

#### 3.2.2 AI literacy (AL)

AI literacy was assessed using the Artificial Intelligence Literacy Scale (AILS), a validated self-report instrument developed to quantify individuals’ competence in engaging with AI systems in everyday tasks (Wang et al., 2023). The AILS defines AI literacy as a four-dimensional construct: (1) awareness of AI, meaning recognition of where and how AI operates in daily and professional contexts; (2) usage, meaning perceived ability to apply AI tools effectively; (3) evaluation, meaning the ability to critically judge the accuracy and limitations of AI outputs; and (4) ethics, meaning responsible and safe use of AI. The original scale was created through item generation, expert review, and psychometric reduction from an initial pool of 65 statements to a final 24 item instrument that demonstrated acceptable structural validity and internal consistency (Lintner, 2024). Each item is rated on a 7-point Likert scale (1 = strongly disagree, 7 = strongly agree). Total scores range from 12 to 84, with higher scores indicating higher AI literacy. Cronbach’s *α* = 0.85 and KMO value = 0.894, supporting its suitability for use in medical education research.

#### 3.2.3 Learning motivation (LM)

Learning motivation was measured using the Academic Motivation Scale, College Version (AMS-C). The AMS-C is a 28-item instrument, which conceptualizes motivation along a continuum from intrinsic motivation to various forms of extrinsic regulation and finally amotivation, reflecting progressively lower levels of self-determination (Vallerand et al., 1992). Items are rated on a 5-point Likert scale ranging from 1 (“does not correspond at all”) to 5 (“corresponds exactly”). Scores range from 28 to 140.

#### 3.2.4 Self-efficacy (SE)

Self-efficacy was assessed using the 10-item General Self-Efficacy Scale (GSES). The GSES has been validated across health and higher education samples and demonstrates strong psychometric properties (Chen et al., 2001; Luszczynska et al., 2005). The GSES captures a learner’s general belief in their ability to handle difficult tasks, persist in the face of challenges, and achieve desired outcomes through their own effort. Each item reflects an individual’s perceived capacity to effectively handle demanding situations (e.g., “I can always solve difficult problems if I try hard enough”), rated on a 4-point Likert scale (1 = strongly disagree, 4 = strongly agree). Higher scores indicate stronger self-efficacy.

#### 3.2.5 Academic performance (AP)

The ophthalmology module is delivered as an intensive block within the clinical curriculum and covers core topics in ocular anatomy and physiology, common anterior and posterior segment diseases, basic ophthalmic examination techniques, and principles of diagnosis and management. Teaching in the module consists of large group lectures, small group case discussions, image-based interpretation exercises, and supervised skills practice. At the conclusion of the course, all students complete a summative final examination that contributes to the course grade. The present research uses this final examination score as an objective index of academic performance in ophthalmology. Scores range from 0 to 100.

### 3.3 Statistical analyses

Data analysis was conducted using Mplus 8.3 and R 4.5.1. First, Harman’s single-factor test and a confirmatory factor analysis (CFA) method were employed to assess common method bias. Descriptive statistics and Pearson correlations were calculated to examine bivariate associations among variables. We then employed structural equation modeling to test the hypotheses. The direct, mediating, and moderating effects were examined using the bootstrapping method with 5,000 resamples to generate bias-corrected confidence intervals. The significance level was set at *p* < 0.05.

## 4 Results

### 4.1 Common method Bias test

To address potential concerns regarding common method bias, we conducted Harman’s single-factor test. The results revealed that the first principal component had an eigenvalue greater than 1 and accounted for 23.58% of the total variance, which is well below the recommended threshold of 40%. Furthermore, we employed a more rigorous approach by adding a common method factor to the original five-factor model (AU, AL, LM, SE, AP). The model comparison showed minimal changes in fit indices between the model with and without the method factor (ΔCFI = 0.016, ΔTLI = 0.013, ΔRMSEA = 0.007). These negligible differences provide additional evidence that common method bias does not substantially affect the interpretation of our results.

### 4.2 Confirmatory factor analysis

This study conducted a confirmatory factor analysis to examine the discriminant validity. We constructed a five-factor baseline model comprising AU, AL, LM, SE, and AP, followed by four alternative nested models for comparison. The results shown in Table 1 demonstrate that the five-factor model exhibited superior fit indices compared to all competing models. These findings confirm adequate discriminant validity among the constructs and support the measurement model’s appropriateness for subsequent structural equation modeling analyses.

**Table 1:** Confirmatory factor analysis.

Model	*χ*^2^/df	CFI	TLI	RMSEA
Five-factor model	2.84	0.96	0.94	0.07
Four-factor model	5.67	0.87	0.86	0.11
Three-factor model	8.34	0.79	0.77	0.14
Two-factor model	11.07	0.73	0.71	0.18
One-factor model	13.39	0.65	0.62	0.20

### 4.3 Descriptive statistics and Pearson correlation coefficient

Table 2 presents the means, standard deviations, and correlations among study variables. Artificial intelligence usage demonstrated significant positive correlations with learning motivation (*r* = 0.22, *p* < 0.01), self-efficacy (*r* = 0.41, *p* < 0.01), and academic performance (*r* = 0.38, *p* < 0.01). Additionally, learning motivation was positively associated with self-efficacy (*r* = 0.30, *p* < 0.01) and academic performance (*r* = 0.28, *p* < 0.01). These correlational patterns provide preliminary support for our theoretical framework.

**Table 2:** Descriptive statistics and correlations among study variables.

Variable	Mean	SD	1	2	3	4	5
1 AI usage	13.98	1.01	1				
2 AI literacy	58.98	1.91	0.18	1			
3 Learning motivation	92.14	3.11	0.22	0.19	1		
4 Self-efficacy	26.95	1.99	0.41	0.27	0.30	1	
5 Academic performance	77.24	3.93	0.38	0.39	0.28	0.16	1

### 4.4 The mediating effects of learning motivation and self-efficacy

**Figure 2:**
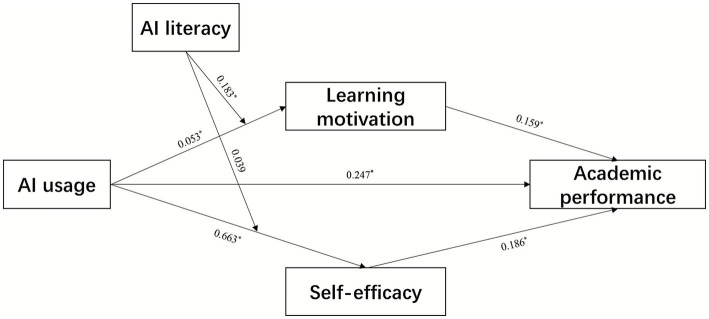
The dual mediating effect of the impact of AU on AP. * represents *p* < 0.05.

In the model, results shown in Table 3 and Figure 2 indicate that AI usage has a significant positive relationship with academic performance (*β* = 0.247, *p* < 0.001). Thus, H1 is supported.

**Table 3:** Results of path analysis.

Path	*β*	SE	*t*	*P*	LLCL	ULCL
AU → AP(direct effect c′)	0.247	0.045	5.489	<0.001	0.158	0.336
AU → LM	0.053	0.009	6.822	<0.001	0.035	0.071
AU → SE	0.663	0.021	2.257	0.024	0.621	0.705
AU × AL → SE	0.039	0.043	0.907	0.364	−0.046	0.124
AU × AL → LM	0.183	0.034	4.159	<0.001	0.115	0.251
LM → AP	0.159	0.013	5.031	<0.001	0.133	0.185
SE → AP	0.186	0.027	7.022	<0.001	0.132	0.240
AU → LM → AP	0.081	0.011	6.184	<0.001	0.059	0.103
AU → SE → AP	0.079	0.031	5.031	<0.001	0.017	0.141
Total indirect (LM + SE)	0.161	0.036	7.139	<0.001	0.089	0.233
AU → AP (total effect = direct + indirect)	0.409	0.041	10.154	<0.001	0.327	0.491

To test the mediating effects of learning motivation and self-efficacy between AI usage and academic performance, bootstrap results from 5,000 resamples yielded standardized indirect effects of 0.081 and 0.079, respectively. Both 95% confidence intervals excluded zero ([0.059, 0.103] and [0.017, 0.141], respectively). Thus, the results indicate that both learning motivation and self-efficacy mediate the relationship between AI usage and academic performance. Consequently, H2a and H2b are supported. Thus, the results indicate that both learning motivation and self-efficacy mediate the effect of AI usage on academic performance. Consequently, H2a and H2b are supported.

### 4.5 Moderated mediation analyses

The results are shown in Table 4. In the model, the interaction term between AU and AL exhibits a significant positive predictive correlation with LM (*β* = 0.183, *p* < 0.001), indicating that higher AL strengthens the positive impact of AU on LM. However, the interaction term between AU and AL did not significantly predict SE (*β* = 0.039, *p* = 0.364), indicating that AL does not significantly alter the strength of AU’s influence on SE. Therefore, H3a is supported, while H3b is not.

**Table 4:** Analysis of the moderating effects.

Variable	AL level	Effect size	SE	LLCL	ULCL
LM	Low (−1 SD)	0.304	0.103	0.098	0.510
	High (+1 SD)	0.213	0.136	−0.059	0.485
SE	Low (−1 SD)	0.481	0.061	0.359	0.603
	High (+1 SD)	0.523	0.136	0.251	0.795

**Figure 3:**
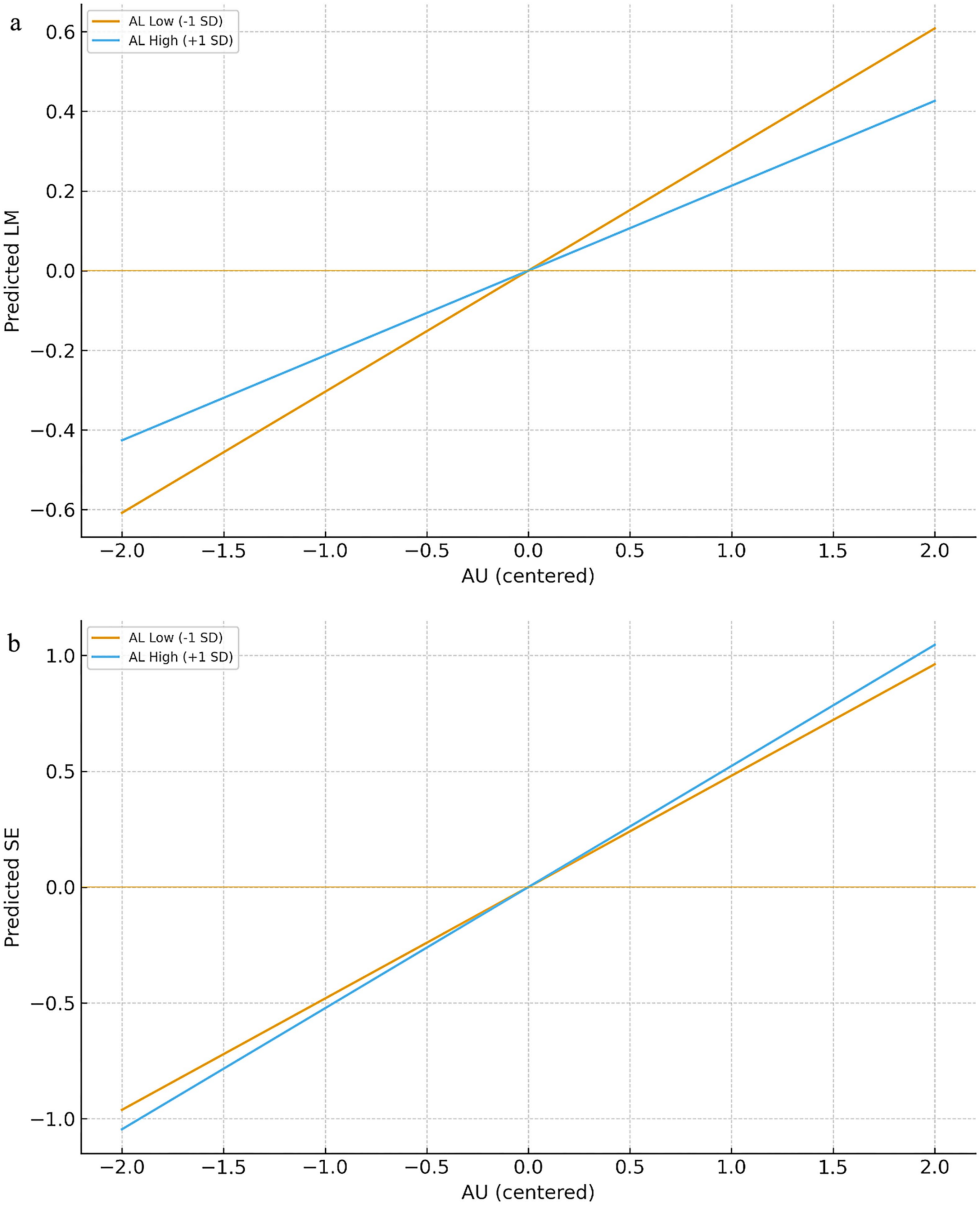
The moderate role of AU. **(A)** The interaction between AU and AL on LM. **(B)** The interaction between AU and AL on SE.

To further validate this hypothesis, we subsequently analyzed the simple slopes of AU for lower AL levels (below one standard deviation from the mean) and higher AL levels (above one standard deviation from the mean), as illustrated in Figures 3A,B. Among students with higher AL levels, AU was positively correlated with AP, whereas among those with lower AL levels, the relationship between AU and AP was weaker. Using a bootstrap procedure with 5,000 resamples, we tested the moderated mediation effects. According to Table 4, when AL was low, the indirect effect via LM was 0.304, with a 95% confidence interval of [0.098, 0.510], which does not include 0. When AL was high, the indirect effect via LM was 0.150, with a 95% confidence interval of [−0.059, 0.485], which includes 0. These findings indicate a significant difference across AL levels for the LM pathway. By contrast, no significant difference was observed for the SE pathway. These findings further support H3a and reject H3b.

## 5 Discussion

The integration of AI into medical education has been widely advocated as a transformative force, with a growing body of literature demonstrating its potential to enhance learning outcomes through personalized feedback, simulation, and accessible resources (Waisberg et al., 2025). However, many existing studies have primarily focused on establishing a direct correlation between AI tool usage and academic performance, often overlooking the underlying psychological mechanisms that translate technological interaction into educational gain. The present study demonstrates that AI usage in an undergraduate ophthalmology module is associated with end-of-course academic performance through the parallel mediating roles of learning motivation and self-efficacy, with AI literacy moderating the relationship between AI usage and learning motivation.

Our results first confirmed a significant direct positive association of AI usage on ophthalmology academic performance (*β* = 0.247, *p* < 0.001, H1 supported). Greater AI usage predicts higher academic performance in ophthalmology, aligns conceptually with recent analyses of LLMs in ophthalmology education, which suggest that generative AI can provide on-demand explanations of board-style questions, generate targeted practice items, and model expert-like diagnostic reasoning for learners (Heinke et al., 2024). Such affordances plausibly enhance individualized study efficiency in visually dense domains like ophthalmology, where mastery often depends on iterative exposure to image-based patterns and clinical decision heuristics.

The parallel mediation results indicate that AI usage may be associated with higher grades partly because it is associated with higher learning motivation and self-efficacy. This is theoretically consistent with self-determination theory, which posits that environments supporting autonomy, competence, and relatedness tend to strengthen autonomous motivation, sustain effort, and improve learning outcomes (Ryan and Deci, 2000). In medical education, motivation has repeatedly been linked to persistence, engagement, and achievement, including evidence that more self-determined forms of academic motivation predict better performance in pre-clinical and clinical coursework (Wu et al., 2020). Our mediation estimates (indirect effect via LM = 0.081) suggest that one pathway from AI usage to good achievement performance runs through heightened learning motivation to learn ophthalmology.

Self-efficacy provides a second, independent pathway. Decades of research in medical and health professions education show that higher academic self-efficacy is associated with greater persistence, deeper cognitive strategy use, and superior academic performance (Artino, 2012). Structural equation models similarly report that self-efficacy is one of the strongest psychological predictors of grades, and that increases in self-efficacy and achievement can reinforce each other over time (Honicke et al., 2023). In our model, AI usage may supply precisely the kind of iterative mastery experiences and explanatory feedback that strengthen ophthalmology-specific confidence (for example, “I can correctly distinguish papilledema from pseudopapilledema on fundus images”), which in turn predicts better exam performance (indirect effect via SE = 0.079). These results extend prior work in medical students showing mediated links between motivational beliefs, cognitive strategies, and grades, by demonstrating that learning motivation and self-efficacy can operate in parallel rather than sequentially, and that both are relevant in a technology-supported ophthalmology module.

A pivotal finding of this study is the differential moderating role of AI literacy, which adds a critical layer of contingency to our model. Significant Moderation on the Path to learning motivation (H3a supported): The significant interaction effect (*β* = 0.183, *p* < 0.001) indicates that the positive impact of AI usage on learning motivation is substantially stronger for students with high AI literacy. The mechanism for this is likely rooted in cognitive load theory and user experience. Students with high AI literacy can navigate the interface and interpret the system’s outputs with less cognitive effort and frustration (Sweller, 2011). This seamless interaction allows them to fully engage with the learning content itself, leading to a more positive and motivating experience. Conversely, students with low AI literacy may expend significant mental resources simply on operating the tool, leading to confusion and anxiety that undermines their intrinsic motivation.

In contrast, AL did not significantly moderate the association between AU and SE (*p* = 0.364), and H3b was therefore not supported. One interpretation is that the kind of self-belief captured by our SE measure reflects a relatively generalized sense of academic capability that is known to be psychometrically stable across cultures (Scholz et al., 2002). General self-efficacy is shaped primarily by mastery experiences, credible feedback, and opportunities to enact skills successfully, rather than by nuanced technical understanding of the learning tool per se (Artino, 2012). In other words, a student can feel “I can learn ophthalmology well enough to do well on the exam” after repeated, AI-assisted practice that yields correct answers and clear explanations, even if that student cannot articulate in detail how the AI generated those explanations. This suggests a potential misalignment in the specificity of the constructs measured: AI literacy reflects a technology-specific competency, whereas the present study assessed general self-efficacy. According to Bandura’s theorizing, self-efficacy is most responsive to influences that operate at a comparable level of specificity (Yan et al., 2025). The relationship between AI usage and self-efficacy might demonstrate greater sensitivity to AI literacy if self-efficacy were evaluated at a domain-specific level, such as within ophthalmology, or in relation to AI-assisted learning tasks. Future research should therefore incorporate domain- or task-specific self-efficacy measures to more precisely examine such contingent relationships. Additionally, moderation may be masked by a specificity mismatch: we measured a broad, trait-like general self-efficacy, whereas AI literacy is a task-proximal, technology-specific competence. Theory and evidence emphasize that self-efficacy is most sensitive to influences at comparable levels of specificity; domain- or task-specific efficacy (for example, fundus-image interpretation efficacy) typically shows stronger contingency than global beliefs, suggesting that any AI-literacy contingent effects would be more detectable on ophthalmology-specific efficacy than on a general scale (Bong and Skaalvik, 2003).

### 5.1 Limitations and future research directions

First, the cross-sectional design, while revealing robust associations, precludes causal inference. The identified relationships and mediating pathways are predictive and theoretically plausible, yet causality cannot be determined. Future studies should adopt longitudinal or experimental designs to better establish causal direction. Second, the use of self-reported measures for AI usage, motivation, and self-efficacy may be subject to biases. The short, self-reported AI usage scale, while practical, may not fully capture the complexity or diversity of AI interactions. Future studies could benefit from incorporating objective metrics, such as backend analytics of actual AI tool engagement or behavioral assessments of performance. Third, the use of a general self-efficacy scale may not be sensitive to changes or influences specific to the ophthalmology learning context or to AI tool use. Future studies should consider using or developing domain-specific self-efficacy measures. Fourth, the study was conducted at a single university in China and focused on ophthalmology, which may limit the generalizability of the findings to other institutions, cultural contexts, or medical specialties. Replication in diverse settings and disciplines is needed.

### 5.2 Implications

Ophthalmology educators should strategically embed vetted AI tools into routine formative practice aligned with image-rich objectives. They should also explicitly teach foundational AI literacy to translate AI engagement into motivational gains. Course design must support autonomy and competence through adaptive quizzes, stepwise image explanations, and rapid feedback, thereby strengthening learning motivation and self-efficacy and improving exam performance. Programs should monitor motivation and self-efficacy as core outcomes, provide structured onboarding for students with low AI literacy to prevent inequities, and invest in faculty development for safe, effective AI pedagogy and assessment, enabling learners to use technology productively in specialized domains.

## 6 Conclusion

This study demonstrates that artificial intelligence usage enhances academic performance in ophthalmology education not only directly but also indirectly through the parallel mediation of learning motivation and self-efficacy. Crucially, AI literacy shapes these processes by amplifying the positive association between AI usage and learning motivation, but it does not significantly alter the path from AI usage to self-efficacy. These findings illuminate the psychological mechanisms through which AI influences academic performance, highlighting that its effectiveness depends on both learning motivational states and efficacy. To maximize effectiveness, curricula should embed vetted AI tools that support motivation and efficacy, pair them with foundational AI literacy instruction, and provide structured formative feedback.

## Data Availability

The raw data supporting the conclusions of this article will be made available by the authors, without undue reservation.
